# Optimizing outcomes from cardiac resynchronization therapy: what do recent data and insights say?

**DOI:** 10.1080/14779072.2024.2445246

**Published:** 2024-12-18

**Authors:** Felicity de Vere, Nadeev Wijesuriya, Sandra Howell, Mark K. Elliott, Vishal Mehta, Nilanka N. Mannakkara, Marina Strocchi, Steven A. Niederer, Christopher A. Rinaldi

**Affiliations:** a School of Biomedical Engineering and Imaging Sciences, King’s College London, London, UK; b Department of Cardiology, Guy’s and St Thomas’ NHS Foundation Trust, London, UK

**Keywords:** Cardiac resynchronization therapy, biventricular pacing, heart failure, conduction system pacing, device-based algorithms, arrhythmia, artificial intelligence, in silico trials

## Abstract

**Introduction:**

Cardiac Resynchronization Therapy (CRT) is an effective treatment for heart failure (HF) in approximately two-thirds of recipients, with a third remaining CRT ‘non-responders.’ There is an increasing body of evidence exploring the reasons behind non-response, as well as ways to preempt or counteract it.

**Areas covered:**

This review will examine the most recent evidence regarding optimizing outcomes from CRT, as well as explore whether traditional CRT indeed remains the best first-line therapy for electrical resynchronization in HF. We will start by discussing methods of preempting non-response, such as refining patient selection and procedural technique, before reviewing how responses can be optimized post-implantation. For the purpose of this review, evidence was gathered from electronic literature searches (via PubMed and GoogleScholar), with a particular focus on primary evidence published in the last 5 years.

**Expert opinion:**

Ever-expanding research in the field of device therapy has armed physicians with more tools than ever to treat dyssynchronous HF. Newer developments, such as artificial intelligence (AI) guided device programming and conduction system pacing (CSP) are particularly exciting, and we will discuss how they could eventually lead to truly personalized care by maximizing outcomes from CRT.

## 1. Introduction

Cardiac Resynchronization Therapy (CRT) is an effective treatment for a large proportion of heart failure (HF) patients with a reduced ejection fraction (EF) and concurrent electrical dyssynchrony [1]. Multiple large trials have demonstrated significant improvements in left ventricular (LV) remodeling, functional status, hospitalizations, and mortality from CRT in this population [1]. However, a significant minority of patients do not achieve benefit from CRT, with a small selection actually worsening [2]. CRT implantation is not without risk, in an already clinically vulnerable, co-morbid population. Thus, it is vital we strive to improve the effectiveness of CRT and reserve such intervention for those most likely to have a meaningful outcome.

**Figure 1.**
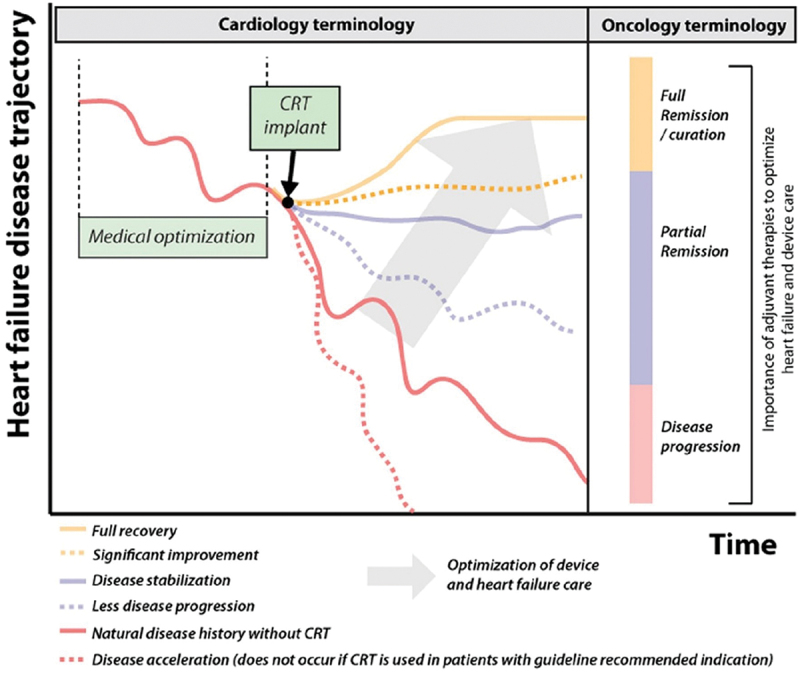
The impact of CRT on HF progression is a spectrum, requiring multimodal intervention to optimize overall outcome. CRT, cardiac resynchronization therapy; HF, heart failure. Reproduced with permission from Mullens et al. [2] under the CC by 4.0 license.

Complicating matters is the inconsistent definition of ‘CRT response’ which varies from echocardiographic measures to symptom assessment. Although a singular definition would be useful for the purpose of consistent trial data, there is also a move toward understanding CRT response as a *spectrum* of outcomes between disease progression and remission (see Figure 1) [2]. For example, some patients may not experience any objective *improvement* post-CRT, but disease stabilization and the avoidance of further disease progression could just as easily be interpreted as a ‘response’ to CRT. Indeed, many argue that the term ‘non-response’ is *‘inherently flawed’* [2], fueling prevalent myths which have resulted in a significant underutilization of CRT in eligible candidates.

Definitions aside, there remains a vast amount of data to decipher on how to maximize potential from CRT. Our article aims to outline the latest evidence for each stage of optimization and the future directions of this ever-expanding field of cutting-edge research.

## 2. Optimizing patient selection

Deciding which patients should be offered CRT is a crucial part of optimizing outcomes. Implanting CRT devices in unsuitable patients introduces unnecessary risk, but equally data indicate its significant underutilization in a large proportion of suitable patients [3,4]. For example, despite data supporting the use of CRT in elderly comorbid populations [5], there remains physician bias against offering procedural intervention to this cohort.

One way to optimize patient selection is through the implementation of a CRT pre-assessment clinic (PAC), which research has shown to be both cost- and clinically effective [6,7]. In our experience, having a CRT PAC provides a holistic ‘one-stop shop’ for an otherwise relatively complex group of patients. It allows for thorough, up-to-date guideline-driven vetting of patients referred for implants and provides a chance to optimize factors that may impair future CRT responses, such as arrhythmia, anemia, and suboptimal medical therapy [8]. Indeed, with ever-improving medical therapies for HF available, it is more important than ever to review a patient’s medication promptly, ensuring they are on guideline-directed medical therapy (GDMT) prior to committing to device therapy. A caveat to this, however, is that we lack large-scale randomized controlled trial (RCT) data on CRT response in the modern HF era, and thus waiting until patients are on all four pillars of GDMT may in fact be delaying definitive treatment. For those unsuitable for CRT, the clinic also offers an opportunity to refer patients on for alternative treatments such as CSP, the merits of which will be discussed later in this review.

With regard to indications for CRT, current guidelines mainly cite research from the original landmark CRT trials conducted over two decades ago [1]. Parameters explicitly stated as indications for CRT include QRS duration (QRSd) and morphology and LV ejection fraction (LVEF), whereas other predictive factors are *mentioned* in the guidelines but absent from final recommendations. These include considerations such as patient sex, HF etiology, and other electrocardiographic (ECG) parameters such as PR interval (PRi) [1]. A greater understanding of the true heterogeneity of CRT candidates and response has prompted efforts to find more robust predictors of outcomes, leading to a surplus of data on how we can enhance the sensitivity and specificity of patient selection.

### 2.1. ECG parameters in patient selection

Due to the proven deleterious effects of CRT in patients without sufficient preexisting electrical dyssynchrony [9–11], international guidelines do not recommend CRT implantation for HF if QRSd is <120 ms [12,13] or <130 ms [1,14]. However, it remains controversial as to whether all HF patients with severe LV systolic dysfunction and a broad QRS should be offered CRT, as demonstrated by significant variations between international recommendations [1,12–14].

Non-LBBB QRS morphology is one such controversy. The evidence for CRT in these patients with QRSd <150 ms is lacking, with consistent findings of no effect [15,16] or even harm [17]. Increasingly apparent is that not all non-LBBB patients respond equally to CRT, even if their QRSd is ≥150 ms. A recent meta-analysis of the landmark CRT trials separately assessed outcomes from CRT in those with either LBBB, right bundle branch block (RBBB) or nonspecific intraventricular conduction delay (IVCD) [18]. It found a similar magnitude of benefit from CRT in those with QRSd ≥150 ms with LBBB (HF hospitalization (HFH) or death: hazard ratio (HR) 0.56; 95% credible interval (CrI) 0.48–0.65) and IVCD (HR 0.59; 95% CrI 0.39–0.89), but no significant benefit in those with typical RBBB (HR 0.97; 95% CrI 0.8–1.34). This suggests a decoupling of RBBB and IVCD as two separate entities when assessing non-LBBB candidates for CRT. The most prominent theory to explain this difference is that IVCD is a form of RBBB with concomitant underlying LBBB, the latter deriving the benefit from CRT. Pastore et al.’s work in 2018 was particularly influential in defining a form of ‘atypical RBBB’ with coexisting LBBB in electrophysiology studies, which they showed translated into better long-term outcomes from CRT for atypical vs typical RBBB patients (echocardiographic CRT response at 2 years: 71.4% vs 19.4%; *p* = 0.001) [19].

There has been similar work exploring the significance of PRi, whereby a lack of sufficient pre-implantation PR prolongation on ECG seemingly stunts future response from CRT. For example, Kutyifa et al.’s subgroup analysis of the MADIT-CRT trial demonstrated a two-fold increase in mortality risk for non-LBBB patients with PRi <230 ms receiving CRT with implantable cardioverter defibrillator (ICD) versus ICD alone (HR 2.14; 95% confidence interval (CI) 1.12–4.09; *p* = 0.022) [20]. In a similar vein, the recent HOPE-HF double-blinded RCT (*n* = 167) found specifically targeting excessive PR prolongation (PRi ≥200 ms) with atrioventricular (AV) optimized His-bundle pacing (HBP) in patients with LVEF ≤ 40%, and either QRS ≤140 ms or RBBB, significantly improved patient quality of life (QOL) and symptoms, with 76% of patients preferring to have pacing switched on rather than off (*p* < 0.0001) [21]. No recent evidence disputes the relevance of preexisting AV dromotropy in predicting CRT response, or the clinical benefit from optimizing PR prolongation, which makes the absence of PRi assessment from international CRT indications perplexing.

### 2.2. The role of artificial intelligence in ECG-based patient selection

**Figure 2.**
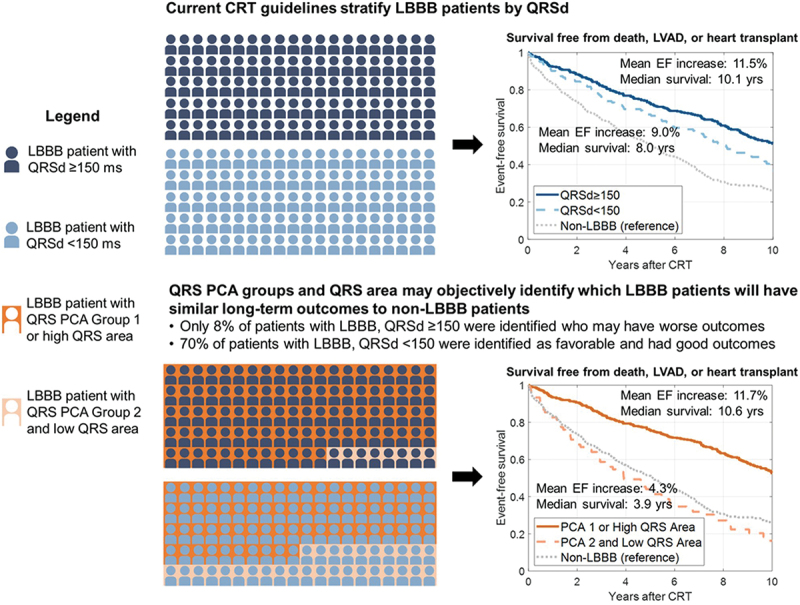
Comparison of CRT patient selection in LBBB patients using QRSd vs QRS PCA and QRS area. CRT, cardiac resynchronization therapy; LBBB, left bundle branch block; QRSd, QRS duration; PCA, principal component analysis. Reproduced with permission from Feeny et al (2020) [25] under the CC BY-NC 4.0 license.

With the latest data suggesting we need more detailed, individualized ECG evaluation in CRT candidate selection, there is a logical move toward automation using artificial intelligence (AI). Specifically, AI can analyze interactions between multiple patient characteristics, including ECG parameters and vectorcardiographic measures such as QRSarea, the latter of which has been shown to predict CRT response more accurately than the conventional measures of QRSd and morphology [22–24]. One recent example used machine learning (ML) to create two main QRS subgroups from the baseline ECGs of 946 CRT recipients [25]. An unsupervised ML method called principal component analysis (PCA) analyzed multiple ECG parameters to identify two main clusters of patients with high (group 1) vs low (group 2) QRS PCA scores. Higher scores were driven by LBBB characteristics such as positive deflections in lateral ECG leads, whereas lower scores occurred when QRS complexes more closely resembled RBBB morphology. Stratifying all patients by QRS PCA group predicted event-free survival following CRT, with group 1 incurring less risk of the composite endpoint of death, LV assist device (LVAD) implant or heart transplantation (HTx) (HR 0.45; 95% CI 0.38–0.53; *p* < 0.001) and a greater improvement in LVEF (+11.1 ± 11.7% vs +4.8 ± 9.7%; *p* < 0.001). Using QRS PCA and QRSarea in conjunction with QRS morphology meant patients could be further stratified; as a result, they were able to identify a large proportion of LBBB patients with QRSd <150 ms with similarly favorable CRT outcomes to patients with traditional class I indication for CRT, and some class I indication patients with less favorable outcomes than might be expected (see Figure 2). This indicates AI-ECG tools can both limit the overuse of CRT in those unlikely to respond as well as broaden its application to underrepresented populations.

### 2.3. Sex differences in ECG parameters

New research has highlighted important differences between male and female ECG characteristics. Specifically, smaller female heart size is thought to be partially why women respond more to CRT, as this renders their level of baseline dyssynchrony relatively higher than men with the same QRSd [26]. A recent meta-analysis of seven pivotal CRT trials investigated whether indexing QRSd to measures of patient and heart size (including height, body surface area (BSA), and LV end-diastolic dimension (LVEDD)) could explain outcome differences between the sexes [27]. Interestingly, although women were indeed shorter in height with smaller BSAs and had approximately 20% less risk of HFH or death than men (HR 0.54 vs 0.77; p(interaction) = 0.009), indexing QRSd to height, BSA or LVEDD did not fully attenuate the relative sex-specific benefit of CRT, meaning this cannot be the only reason women benefit more from CRT than men.

Other trials have used ‘QRSd/LVEDV’ (LV end-diastolic volume) across both sexes as a form of ‘modified QRSd’ in retrospective analyses of clinical outcome data [28]. Yamamoto et al.’s analysis of 119 CRT recipients with mid-range QRSd 120–150 ms found that having a modified QRSd of ≥0.65 ms/ml was significantly associated with a reduced risk of HFH (HR 0.46; 95% CI 0.25–0.86; *p* = 0.01) [29]. Zweerink et al. had similar findings, where a baseline modified QRSd <0.52 ms/ml increased risk of death, LVAD implant, or HTx significantly (HR 2.13; *p* = 0.001) [30].

Thus, both heart size and sex appear to independently influence CRT outcomes, but heart size does not solely account for sex-driven responses. Of note, many of these trials were performed in exclusively nonischemic cohorts or included a relative minority of ischemic patients; this is relevant as nonischemic cardiomyopathy is another independent predictor of CRT outcome and is more prevalent in women than men [30,31].

### 2.4. Imaging parameters in patient selection

As well as electrocardiography, imaging parameters such as echocardiography and cardiac magnetic resonance (CMR) can be used to predict outcomes from CRT. Currently, LVEF is the only imaging parameter used as a cutoff in international CRT and ICD guidelines [1,32]. However, recent evidence has prompted moves toward a more sophisticated use of imaging in the CRT referral process.

One example of this is echocardiographic mechanical dyssynchrony, which has yielded mixed results over the years. Initially, single-center trials were very positive about the predictive capability of mechanical dyssynchrony for CRT outcomes [33,34]. However, when the multi-center EchoCRT trial implanted devices in *narrow QRS* patients with mechanical dyssynchrony, CRT actually caused harm, and the trial had to be stopped early for futility [11]. Prior to this, the multi-center PROSPECT trial found only modest, variable predictive value from 12 different markers of mechanical dyssynchrony when prospectively analyzing the echocardiograms (in a blinded core laboratory) of 426 patients who received CRT for standard broad QRS HF indications [35].

Recent observational data have been more supportive of this echocardiographic parameter [36]. In one retrospective analysis of 1060 CRT patients, apical rocking and septal flash were found to be significantly more predictive of CRT response and all-cause mortality than standard international guidelines at the time (log-rank *p* < 0.0001, 0.006 & 0.004 vs Class I, IIa & IIb recommendations, respectively) [37]. Interestingly, the addition of these dyssynchrony parameters to European guidelines in the pre-assessment of this cohort improved the sensitivity of CRT response prediction to such an extent that even those with class III (meaning ‘not recommended’) indication for CRT with mechanical dyssynchrony on echocardiography had similar CRT response levels to those with a Class I indication (Class III + dyssynchrony: 18/26 responders (69%) vs Class I overall 256/389 (66%) responders; no statistical comparison available). This result is in direct contrast to the EchoCRT trial [11], further confusing the relative importance of mechanical dyssynchrony in CRT pre-assessment.

CMR offers valuable information that we can use to predict who will benefit from CRT. In particular, the burden and location of myocardial scar (most notably posterolateral and septal scar) detected by CMR has already been shown to be predictive of CRT outcomes [38–40]. More recently, CMR data has been combined with echocardiographic data to further enhance the sensitivity of CRT response prediction. In a prospective study of 170 CRT recipients, assessments of septal scar by CMR and mechanical dyssynchrony by echocardiography were combined to predict CRT response, defined as ≥15% reduction in LV end-systolic volume (LVESV) at 12-month follow-up [39]. They found that the addition of septal scar significantly improved the already significantly predictive power of mechanical dyssynchrony (*p* = 0.003, <0.0001 and 0.007 when comparing three separate dyssynchrony parameters in isolation versus in combination with septal scar), with anterior, inferior, and lateral wall scars all lacking the same predictive capability individually. Of note, the absence of septal scar remained an independent predictor of CRT response (area under the curve (AUC) 0.67; 95% CI 0.52–0.83; *p* < 0.05).

Similar to AI-ECG, imaging parameters have been incorporated into ML models to predict CRT outcomes. Puyol-Antón et al. aimed to predict CRT outcomes using multimodal deep learning, a subset of ML which uses advanced computing to create an artificial neural network capable of incorporating information from multiple sources (in this case multiple imaging modalities), with little human input required [41]. Their multimodal approach, which combined echocardiographic and CMR data, was able to predict CRT response with 83% sensitivity and 71% specificity, which is comparable to current leading examples of imaging-based CRT response prediction [39,42,43]. The major advantage, however, is the fact that the model was fully automated, requiring far less manual intervention than any of the other AI tools cited in this review. They also proved predictive value from both echocardiography and CMR in isolation using the model, making it relevant to the majority of real-world patients who usually only undergo a single imaging modality pre-implant.

### 2.5. Novel biomarkers predicting CRT response

Another emerging field is the search for novel biomarkers capable of predicting CRT response. For example, Gambardella et al. found that low baseline levels of Ryanodine Receptor 1 (RyR1) glycation independently predicted CRT response at 1 year, as defined as a > 15% reduction in the indexed LVESV (*p* < 0.0001) [44]. In another recent study, baseline levels of microRNA-130, a molecule downregulated in endothelial dysfunction, were predictive of the echocardiographic CRT response (HR 1.490; CI 95% 1.014–2.188; *p* = 0.042) [45]. The same group also found that higher levels of microRNA-30 (which is thought to be involved in multiple aspects of ventricular remodeling) [46,47] were predictive of the response from left bundle branch pacing (LBBP) (HR 2.713; 95% CI 1.543–4.769; *p* = 0.001), an alternative treatment to conventional CRT which will be discussed later in this review [48]. Not only could these biomarkers play a role in predicting CRT response, but they also help us understand some of the cellular and epigenetic mechanisms behind it [45]. Practically, one may envisage their inclusion in future ML tools used to decide suitability for CRT, or even consider them as potential therapy targets [47,49].

## 3. Optimizing CRT implantation

Once it has been decided that a patient needs CRT, the exact method of implantation requires consideration. Conventional CRT (cCRT) involves the co-implantation of an endocardial right ventricular (RV) pacing lead and an epicardial LV pacing lead, which together resynchronize mechanical LV contraction. However, the significant rates of CRT non-response and limitations of epicardial LV lead placement have prompted discussions about alternative methods of resynchronization therapy. Current options include optimizing epicardial LV lead placement (e.g. with image-guidance or using multiple leads) or choosing a different lead position altogether, the latter of which has gained significant traction in recent years.

### 3.1. Optimizing epicardial LV lead placement

We already know that the presence of LV scar is predictive of poorer CRT outcomes [38,39] and that scar tissue cannot adequately propagate pacing impulses [50]. Thus, a logical approach to optimizing CRT is to position LV leads away from the scar. In addition, researchers have also considered targeting areas of late mechanical activation (LMA), as theoretically this should provide the greatest improvement in electrical synchrony from baseline [51,52]. Retrospective observational trials such as Taylor et al.’s have shown that targeting areas of LMA predicts LV reverse remodeling (LVRR) (*p* < 0.001) and that avoiding scar predicts cardiac mortality (adjusted odds ratio (OR) 0.27; 95% CI 0.12–0.62) [53]. However, translating this into clinical practice has proven exceptionally challenging, as the procedural limitations of coronary vein anatomy, lead stability, and phrenic nerve stimulation significantly impair an operators’ ability to pre-specify final lead position. In the STARTER trial [52], these factors led to only 30% of patients having LV leads positioned in the image-guided target segment, and there was only 52% LV lead concordance in the more recent CMR-CRT trial [54]. Thus, although using pre-procedural imaging to target scar-free areas of LMA is attractive in theory, image-guided epicardial LV lead placement has significant constraints in real-world practice.

Another option is pacing the LV from multiple sites, which theoretically increases the chance of reaching favorable areas of cardiac tissue and provides the ability to stimulate a larger portion of myocardium to achieve more rapid electrical activation [55]. Multisite pacing can be delivered either through a single quadripolar LV lead capable of multipoint pacing (MPP) or through the use of multiple LV leads. A few individual trials have reported benefit from multisite pacing, for example, one RCT focusing on diabetic patients reported significant improvements in HFH rates (15.2% vs 25%, *p* = 0.046) and arrhythmic events (7% vs 16.7%, *p* = 0.019) in those receiving multipolar pacing vs cCRT, but no significant improvement in CRT response rates (61.6% vs 58%; *p* = 0.27) [56]. When reviewing seven MPP trials, one large meta-analysis demonstrated significant improvements in LVRR (OR 5.33; 95% CI 3.05–9.33; *p* < 0.001), but no significant impact on QOL parameters (clinical composite score (CCS) change ≥ 1: OR 2.37; 95% CI: 0.68–8.28; *p* = 0.178 and New York Heart Association (NYHA) class change ≥ 1: OR 2.49; 95% CI 0.74–8.42; *p* = 0.141) and when non-randomized trials were excluded, there was no longer a significant difference in LVRR (OR 1.96; 95% CI 0.69–5.55; *p* = 0.20) [57]. A similar meta-analysis of six multi-*lead* pacing (i.e. the co-implantation of three ventricular leads, either as two RV leads or two LV leads) RCTs also failed to demonstrate significant improvements in LVRR (mean difference (MD) −0.54 mL, *p* = 0.93), LVEF (MD 1.42%, *p* = 0.40), or mortality (OR 1.11; *p* = 0.77) [58]. The lack of consistent supporting evidence means multisite pacing has yet to see much uptake in clinical practice.

### 3.2. LV endocardial pacing

Until now, our focus has been on epicardial LV pacing (LVP). However, there have been small observational studies examining endocardial LVP, both lead-based and leadless. The main advantages of endocardial LVP are the main disadvantages of epicardial LVP – namely the anatomical limitations of coronary sinus lead placement and its ability to deliver more physiological ventricular activation. Mechanistically, endocardial LVP has shown promise, with multiple studies reporting greater acute hemodynamic response from temporary endocardial versus epicardial pacing [59–61]. However, in practice, lead-based endocardial LVP is associated with a significant stroke risk mandating lifelong anticoagulation, and thus leadless alternatives have since been explored.

RV leadless pacing has already been embraced worldwide, especially in populations with higher risks of lead and pocket-based complications [62]. For those requiring CRT, leadless LV systems are now available and can be used in combination with either conventional endocardial or leadless RV pacemakers, the latter resulting in a completely leadless device [63]. With regard to clinical outcomes, a recent meta-analysis of 181 patients receiving leadless LVP for CRT in five observational trials reported significant increases in LVEF from baseline (MD + 6.3%; 95% CI 4.35–8.19; *p* > 0.001) and LVRR (with variable definitions between trials) in 54% of the patients [64]. Recent data from the prospective SOLVE-CRT trial was also promising, demonstrating a significant reduction in LVESV at 6 months (−16.4%; 95% CI −21.0% to −11.7%; *p* = 0.003) in 183 leadless CRT recipients [65]. However, 19.1% of patients encountered a device or procedure-related complication, likely related to the novel nature of implantation; in addition, the device has a relatively low average battery life of 4.5 years, impacting its cost-effectiveness [66]. Without a head-to-head comparison of leadless CRT vs cCRT, there is no current evidence leadless LV pacing should be used first-line over cCRT unless there are specific patient circumstances that preclude endovascular device implantation.

### 3.3. Conduction system pacing

Impossible to ignore is the rise of conduction system pacing (CSP). Theoretically, CSP provides the most physiological form of pacing by targeting the native conduction system, making it an attractive alternative to conventional epicardial biventricular pacing (BiVP). LBBP, in particular, has emerged as a reliable CSP technique that overcomes many of the procedural hurdles of its predecessor, HBP [67,68]. Thus far, given the relative novelty of CSP as a form of CRT, there are very few published trials directly comparing CSP to BiVP, with heterogeneity in CSP technique used, recruitment criteria, and trial design. However, whilst recognizing the relative paucity of RCT-level data in this field, recently published international guidelines have awarded CSP an equivalent level of recommendation to BiVP for patients with high pacing burden (>20%) and moderately reduced or preserved LVEF, indicating the current strength of support for CSP as a form of resynchronization therapy [69].

Much of the current evidence for CSP in HF patients has come from large retrospective observational trials. One such study compared clinical outcomes between 981 BiVP vs 797 LBBP recipients, all of whom had LVEF ≤ 35% at baseline [70]. LBBP patients experienced significantly greater improvements in QRS narrowing (128 ± 19 ms vs 144 ± 23 ms; *p* < 0.001) and LVEF (+13% ± 12% vs +10% ± 12%; *p* < 0.001) compared to the BiVP group, and significantly less death or HFH over 5 years (HR 1.495; 95% CI 1.213–1.842; *p* < 0.001). Similar results were found by a retrospective sex-specific analysis of 575 female and 1203 male CRT recipients, where women with LBBP experienced a significant reduction in death or HFH over 5 years compared to those with BiVP (HR 0.64; 95% CI 0.43–0.97; *p* = 0.03), whereas male patients did not yield the same benefit (HR 0.88; 95% CI 0.9–1.42; *p* = 0.29) [71]. This is partially explained by a higher proportion of nonischemic vs ischemic cardiomyopathy among the women included, with no impact on death or HFH from LBBP vs BiVP in women with ischemic cardiomyopathy (HR 1.30, 95% CI 0.65–2.62; *p* = 0.45). The fact that female sex was an independent predictor of improved outcome from LBBP, however, would indicate there are other factors at play, such as greater mechanical dyssynchrony per baseline QRSd in women, as discussed previously in Section 2.2.

An important caveat to note from these retrospective trials is that they do not touch upon procedural success rates from CSP, which remain relatively low compared to conventional BiVP in clinical trials [72,73]. This is perhaps to be expected of any new technology and should improve with increasing operator familiarity. The multicenter MELOS study reported on the learning curve with LBBP, showing it took approximately 100 cases before operators experienced plateaus in fluoroscopy time and overall procedural success rates, which remained relatively lower for HF (82.2%) vs bradyarrhythmic (92.4%) indications [74]. The reason for this particular observation has been postulated to be due to higher incidences of LV cavity dilatation and septal fibrosis in HF patients [75,76], which both render CSP lead placement more challenging.

In a prospective observational study of 128 vs 230 patients undergoing LBBP vs BiVP, procedural success was significantly lower among patients undergoing LBBP vs BiVP (84.4% vs 94.7%; *p* = 0.002) [73], which is comparable to the MELOS study [74] when considering only HF patients with LVEF ≤ 35% were included. In their subanalysis of the 338 patients with successful implants, HFH rates were significantly better for LBBP vs BiVP recipients (HR 0.379; 95% CI 0.215–0.699; *p* = 0.001). Significantly greater LVEF improvement from LBBP vs BiVP was also reported (+8.04 ± 9.9% vs +3.9 ± 7.9%; *p* < 0.001), but it was important to note that this was not similarly stratified by procedural success.

With regard to RCT-level data, two small trials have demonstrated noninferiority from HBP over BiVP in HF patients. The ‘His-SYNC’ trial randomized 41 patients meeting CRT indication to receive either HBP or BiVP [77]. Both groups demonstrated significant improvements in LVEF at 6 months (both *p* < 0.001), but HBP did not perform superiorly to BiVP (+9.1% vs +5.2%, *p* = 0.33). Of note, there was a relatively high crossover (48% − 10/21) of those assigned HBP to the BiVP group. This was most commonly due to an inability to reach a preset target QRS narrowing of >20% to a QRS duration of ≤130 ms. The authors postulate that this was related to 50% of HBP crossover patients having baseline IVCD, as previous studies have shown this to be less amenable to HBP than LBBB morphology [78].

Subsequently, the ‘His-Alternative’ trial randomized 50 HF patients (LVEF ≤35%) exclusively with LBBB to receive either HBP or BiVP [72]. In the intention-to-treat analysis, there was no significant difference between treatment groups in the degree of improvement in LVEF (*p* = 0.27), QRS shortening (*p* = 0.51), NYHA class (*p* = 0.56) or LVRR (*p* = 0.55) at 6 months, but both treatment groups showed significant improvements in all parameters from the baseline (*p* < 0.001 for all). In the per-protocol analysis, HBP did outperform BiVP with regard to LVRR at 6 months (LVESV relative difference 44 ± 16% vs 35 ± 15%; *p* = 0.049), but these results should be approached with caution given these patients were not fully matched for baseline characteristics. In addition, pacing thresholds at 6 months were significantly higher in those who received HBP versus BiVP (2.3 ± 1.4 V vs 1.4 ± 0.5 V; *p* < 0.01). This was the main reason for their 28% crossover and is an important factor to consider regarding the feasibility of HBP long term, as it has significant implications for battery longevity and lead integrity.

With regard to LBBP, the recent LBBP-RESYNC RCT compared LBBP to BiVP in 40 nonischemic cardiomyopathy patients (LVEF ≤40%) with LBBB at baseline (with QRSd >140 ms for men or >130 ms for women) [79]. In this study, more patients crossed over from BiVP (4/20–20%) than from LBBP (2/20–10%), which, when compared to the HBP implant failure rates in previously discussed trials, supports LBBP as a relatively more robust method of CSP. In both the intention-to-treat and per-protocol analyses, LBBP achieved a greater improvement in LVEF at 6 months compared to BiVP (intention-to-treat MD 5.6%; 95% CI 0.3%−10.9%; *p* = 0.039, per-protocol MD 7.5%; 95% CI 2%−13%). These are promising results from a relatively small RCT in favor of LBBP over BiVP. Larger studies with longer follow-up periods are required, but from the currently available data, LBBP appears to be a robust, viable alternative to BiVP for HF patients with LBBB requiring CRT.

### 3.4. CSP in patients with atrial fibrillation

CSP has also been shown to be effective in patients with atrial fibrillation (AF) undergoing AV node ablation (AVNA), which is another relative indication for CRT over standard RV pacing (RVP) [1]. In a crossover RCT of 50 patients with AF and LVEF ≤ 40% undergoing AVNA, patients were implanted with both HBP and BiVP, before being randomized to receive either HBP or BiVP for 9 months, and subsequently switching to the other pacing mode for further 9 months [80]. The trial demonstrated a small but statistically significant improvement in LVEF from HBP vs BiVP during the second trial phase (LVEF difference between HBP and BiVP +5.9%; 95% CI 1.2%–10.7%; *p* = 0.015), with no significant difference between HBP and BiVP for any of their secondary outcomes (including LVRR (*p* = 0.279), NYHA class (*p* = 0.317) and B-type natriuretic peptide (BNP) level (*p* = 0.727)). Interestingly, compared to the previously discussed HBP trials, all 50 patients had successful HBP and BiVP implant procedures, with comparably stable LV and HBP lead thresholds at 18-month follow-up. The authors believe this was due to (a) using experienced HBP operators, (b) specifically targeting the *distal* His-bundle and (c) the fact these were patients with a narrow QRS at baseline.

Indeed, the anatomical proximity of the AV node to the His-bundle may be a reason for operators to choose LBBP over HBP in permanent AF patients undergoing AVNA. Thus far, there has been no dedicated trial of LBBP for patients with AF, but a previous observational study of patients with HF and LBBB showed similar improvements in LVEF and NYHA classes between AF and sinus rhythm patients receiving LBBP (no statistical comparison provided) [81]. Intriguingly, in a recent propensity score-matched study of 1414 patients with EF ≤ 35%, LBBP was shown to be less arrhythmogenic than BiVP, conferring a reduced incidence of new-onset AF lasting >30 seconds (2.8% vs 6.6%; *p* = 0.008) or >24 hours (0.7% vs 2.9%; *p* = 0.015), as well as a reduced risk of sustained ventricular arrhythmia (HR 0.46; 95% CI 0.29–0.74; *p* < 0.001) and ICD shock (3.3% vs 7.2%; *p* < 0.001) [82]. Thus, implanting an LBBP system instead of a BiVP device may actually reduce the burden of future arrhythmia in HF patients.

### 3.5. CSP-optimized CRT

CSP is not a suitable treatment for all patients with indications for CRT; for example, CSP is less effective at restoring lateral wall activation in patients with distal LBBB versus proximal LBBB [83]. In addition, the presence of septal scar makes LBBP implantation challenging [84], and in one in silico trial it was found to render CSP ineffective [85]. Septal scar patients also respond relatively poorly to BiVP, making their CRT options relatively limited [39].

**Figure 3.**
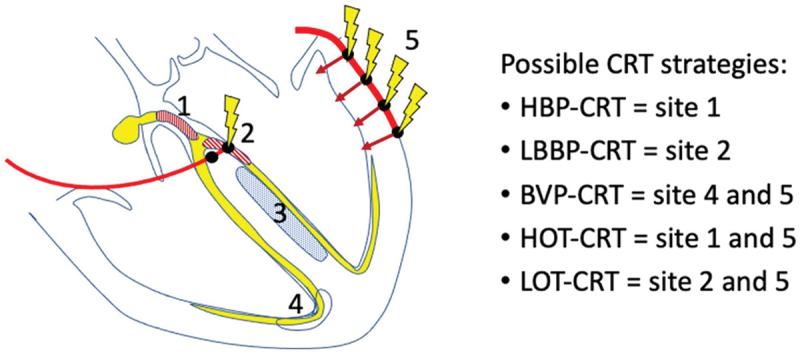
Different types of CSP lead position, including combinations with BVP known as ‘Optimized-CRT.’ BVP, biventricular pacing; CRT, cardiac resynchronization therapy; CSP, conduction system pacing; HBP, His-bundle pacing; HOT-CRT, His-Optimized CRT; LBBP, left bundle branch pacing; LOT-CRT, left bundle branch-Optimized CRT. Reprinted from Vijayaraman P, Chelu MG, Curila K, et al. Cardiac conduction system pacing: a comprehensive update. JACC Clin Electrophysiol. 2023;9(11):2358–2387. doi:10.1016/j.Jacep.2023.06.005 [86], with permission from Elsevier.

To overcome such hurdles, the combination of CSP with BiVP has been investigated, in the form of either His-Optimized CRT (HOT-CRT) or LBBP-Optimized CRT (LOT-CRT) (see Figure 3) [86]. This could be particularly useful for cCRT non-responders, who could have a single additional CSP lead placed in an attempt to maximize electrical resynchronization.

Following on from a successful feasibility cohort study [87], Vijayaraman et al. recently randomized 100 patients indicated for CRT to receive either HOT-CRT or BiVP [88]. Among the 48 patients with a successful HOT-CRT implant, QRSd narrowed from 164 ± 26 ms to 137 ± 20 ms (*p* < 0.001) and in their intention-to-treat analysis, LVEF improved to a greater extent in the HOT-CRT group vs BiVP group (+12.4% ± 7.3% vs +8.0% ± 10.1%; *p* = 0.02). Also, although there was no statistically significant difference between the echocardiographic response rates (80% for HOT-CRT vs 61% for BiVP; *p* = 0.06), 80% still signifies an impressive response rate when compared to the historically reported CRT outcomes [2].

LOT-CRT has shown comparable results; Feng et al. recruited 21 patients indicated for CRT, assigning 11 to BiVP and 10 to LOT-CRT [89]. Interesting to observe in the LOT-CRT group was the sequential reduction in QRSd with different pacing modes; from a mean baseline of 158.0 ± 13.0 ms, QRSd was reduced to 132.0 ± 4.5 ms (*p* = 0.019) by BiVP, 123.0 ± 5.7 ms (*p* < 0.01) by LBBP, and 121.0 ± 3.8 ms by LOT-CRT (*p* = 0.001 compared to BiVP, non-significant when compared to LBBP). Both groups demonstrated improvements in LVEF and BNP at 9-month follow-up compared to the baseline (both *p* < 0.01), but the LOT-CRT cohort outperformed BiVP recipients significantly (both *p* < 0.01). A larger multicenter study of 112 patients with indications for CRT observed similarly positive findings following LOT-CRT; mean QRSd reduced from 182 ± 267 ms to 144 ± 22 ms with LOT-CRT, vastly outperforming BiVP (QRSd 170 ± 30 ms; *p* < 0.001) and LBBP (162 ± 23 ms; *p* < 0.001) [83]. At a mean follow-up of 7.8 ± 2.3 months, significant improvements in LVEF and NYHA classes (both *p* < 0.0001) were also observed.

Preliminary data are extremely promising for optimized-CRT. However, it seems unlikely that it will be made available beyond cCRT and CSP non-responders anytime soon, unless large RCTs show it to have an extraordinary level of benefit that can justify its longer procedure time, relative complexity, and higher cost.

## 4. Optimizing CRT response post-implant

Although the majority of recent data have been investigating decision-making pre- and peri-implant, optimizing outcomes post-implant has been a topic of CRT research since its conception. Most recently, research has focused on automated device technology, but more traditional forms of post-CRT care such as echocardiography-based device optimization and medication management remain relevant to discuss.

### 4.1. Optimizing device settings after CRT

The traditional mainstay of post-CRT optimization is the use of ECG and echocardiographic measures to guide optimal device AV delay (AVD) and VV delay (VVD), which aims to maximize ventricular filling, LV synchrony, and subsequent cardiac output. There is plentiful evidence for these methods achieving acute hemodynamic benefit [90,91], but reproducible data demonstrating a long-term impact on clinical outcomes has been sorely lacking [92].

One issue with device optimization trials is the limited room for further gain on top of initial CRT benefits. For example, in the SMART-AV trial, although neither ‘SmartDelay’ algorithmic-guided nor echocardiography-guided AVD optimizations were superior at improving LVRR or QOL over fixed empirical settings, the fixed AVD of 120 ms was already providing some level of benefit at 6 months (median LVESV reduction −15 ml (range −45 ml to +6 ml), median LVEF improvement +5.1% (range −1.0% to +13.1%)) [93]. Additionally, in the BRAVO trial, 70% of patients were found to have an optimal AVD (via echocardiographic or hemodynamic measurement) within only 20 ms of the nominal setting of 120 ms [94]. AV and VV delay optimizations are thus more likely to benefit those who have not already responded to empirical settings, but only a few small trials dedicated to CRT non-responders are available to support this hypothesis. Brown et al.’s trial used QRS area to guide optimal AV and VV delay in 39 CRT echocardiographic non-responders (defined as ≤5% improvement in LVEF) or ‘incomplete responders’ (defined as LVEF ≤ 40% at least 3 months post-CRT) [95]. Several patients were also switched from BiVP to LV-only pacing (which uses an internal algorithm to co-ordinate LVP with native RV conduction) or vice versa. As a result of their optimization method, LVEF and LVESV both significantly improved over a 6-month follow-up period (LVEF +4.5 ± 5.9%; *p* < 0.001, LVESV −10.5 ± 23.8 ml; *p* = 0.009). Naqvi et al.’s case series of eight CRT non-responders yielded similarly positive outcomes [96], but another small echocardiography-based trial found no significant impact of AV and VV delay optimization on LVEF or LVRR in CRT non-responders at 3-month follow-up (*p* = 0.140 and 0.82 respectively) [97]. Going forward, larger RCTs are required to prove any benefit from traditional echocardiography-based optimization to support its continued use in post-CRT management.

Another hypothesis for why traditional device optimization has yet to prove particularly impactful is the dynamic nature of cardiac conduction. Altering device settings in a one-off clinic visit is unlikely to have a sustainable impact if there is ongoing cardiac remodeling; indeed, we have evidence that optimal AV and VV delays can change significantly over time [98,99], supporting the need for periodic reassessment. We also know that intrinsic AV conduction alters in response to exercise and autonomic tone and can vary significantly beat-to-beat [100,101].

Thus, the ideal method of optimization would be a device-based algorithm that adapts to changes in cardiac conduction in real-time. Such technology is now available from most major device manufacturers and is a hot topic for CRT research. We already mentioned that the ‘SmartDelay’ algorithm had no significant impact on clinical outcomes in the SMART-AV trial when compared to an empiric AVD [93], but it is important to highlight that an updated RCT of this technology tested against a fixed AVD did demonstrate greater improvements in LVEF (46.7% vs 32.1%; *p* = 0.050) and LVESV (−41 ml vs −33 ml; *p* = 0.01) at 6 months when specifically studying patients with relatively long VV delays post-implant [102]. The ‘SyncAV’ algorithm has also been shown to reliably reduce electrical dyssynchrony beyond nominal CRT settings [103,104], but the results of an updated trial are awaited to see if this translates into long-term clinical outcomes [105]. Similarly, another study investigating clinical outcomes from the ‘AutoAdapt’ algorithm is due to complete recruitment this year [106].

By contrast, the ‘AdaptivCRT’ algorithm has already undergone large-scale clinical testing. Adaptive CRT (aCRT) aims to fuse LV stimulation with intrinsic RV conduction, as guided by intrinsic AV intervals (unless there is pathological AVD or atrial arrhythmia). Promising data from an RCT of 180 CRT recipients has already shown that achieving intrinsic RV and paced LV ‘fusion’ at time of implant significantly improves LVRR at 12 months compared to standard BiVP settings (OR 2.02; *p* = 0.026) [107]. However, it is important to note that there was no significant change in clinical response (defined as >10% increase in 6-minute walk test (6MWT) or a 1 step decrease in NYHA class) (OR 1.43; 95% CI 0.79–2.59; *p* = 0.24), and mean QRSd at baseline was exceedingly high (181 ± 22 ms in fusion group, 180 ± 21 ms in control group) compared to most CRT trials.

The Adaptive CRT trial conducted over a decade ago only demonstrated non-inferiority of aCRT versus echocardiography-based CRT optimization [108]. However, subsequent sub-studies of patients with exclusively normal baseline AVDs have demonstrated significant improvements in clinical outcomes, especially when receiving a high proportion of synchronized LVP (sLVP – also known as ‘LV-only pacing’) via the aCRT algorithm. A recent example compared 70 patients from the original treatment group with normal baseline AV interval and ≥80% sLVP to 91 patients from the BiVP control group with normal baseline AV interval. At 12 months, the ≥80% sLVP group had a greater improvement in LVEF when compared to the BiVP group (+8.5 ± 11.3% vs +5.5 ± 10.3%; *p* = 0.038) and greater improvements in global LV radial strain (+6.3 ± 8.6% vs +4.0 ± 10.1%, *p* = 0.046), especially within septal and anteroseptal segments [109]. The authors postulate that this was due to a reduced burden of RVP, which is known to be associated with an increased risk of pacing-induced cardiomyopathy and adverse LV remodeling [110].

Most recently, we had the results of the AdaptResponse trial, a much larger international multicenter RCT of 3617 patients undergoing CRT for conventional HF indications [111]. Interestingly, by having sex-adjusted inclusion criteria for QRSd (≥140 ms for men, ≥130 ms for women), a relatively high proportion of participants were women (43.4%), usually underrepresented in CRT trials. Overall, after a 5-year follow-up, there was no significant reduction in the composite of all-cause mortality and intervention for HF decompensation from aCRT vs cCRT (HR 0.89; 95% CI 0.78–1.01; *p* = 0.077). However, both groups had relatively low mortality rates compared to previous CRT trials (aCRT group 15.6% vs cCRT group 17.4%), including in those with NYHA classes III−IV. Including a higher proportion of women is likely to have influenced this, given data consistently show women achieve better CRT outcomes than men [112,113]. One might also have expected to credit a higher uptake of guideline-directed medical therapy (GDMT) than in older trials, but disappointingly, only 6.4% of participants were taking all four GDMT drug classes, and only 0.5% were on a sodium-glucose co-transporter-2 (SGLT2) inhibitor. Still, these low event rates signal a future trend in CRT trials conducted in the modern medical era, making it harder to prove mortality benefits without very large trial cohorts. Indeed, the continuing downtrend in cardiac mortality has already prompted the redoing of multiple landmark ICD trials for this very reason [114–116].

### 4.2. Device considerations post-CSP implant

Unlike advances in CSP leads, there has been a relative lag in the development of CSP-specific devices, with leads being attached to BiVP devices in the interim. This, alongside the novelty of CSP, has meant there are currently only small-scale observational or experimental studies specific to device optimization post-CSP.

One in silico trial using digital heart models created from 24 HF patients with LBBB examined the effects of different pacing modes and AVDs on ventricular activation. They found that by optimizing AVD during LBBP, RV activation time could be reduced (104.7 ± 8.7 ms vs 141.3 ± 10.0 ms), translating into significant reductions in QRSd (*p* < 0.05) and biventricular dyssynchrony (*p* < 0.05) [117]. This was because optimizing AV conduction meant RV activation could occur via the native right bundle branch (if intact), rather than waiting for propagation across the septum, thus correcting the RBBB-like activation that can occur with LBBP. Similar findings have been demonstrated in vivo, including by Ali et al. [118]. However, in their study, only 6/19 patients (32%) experienced acute hemodynamic benefit from AVD optimization during temporary LBBP, and thus the clinical significance of achieving fusion between LBBP and intrinsic right-bundle conduction remains unclear.

In practice, optimizing QRSd on a 12-lead ECG through altering AVD and other device settings during pacing clinic follow-up is a logical approach post-CSP implant. In addition, given the lack of long-term data on CSP lead stability, we would recommend regular checks of lead threshold and sensing to ensure there has been no micro- or macro-displacement of leads.

### 4.3. Optimizing medical therapy after CRT

Optimizing GDMT is a key part of HF management. Although it is advised to up-titrate GDMT to its maximum dose prior to considering referral for CRT, this is often limited by bradycardia and hypotension in HF patients. When combined with variable access to specialist HF services, this manifests as a disappointingly low level of full adherence to GDMT in clinical practice [119]. In addition, HF patients with LBBB appear to achieve relatively less LVRR from GDMT than average [120], and given they respond more to CRT than non-LBBB patients [15], there is an argument for offering them CRT earlier than the current pre-CRT pathways recommend.

Encouragingly, CRT’s ability to counteract bradycardia and acutely improve blood pressure has been shown by multiple studies to facilitate higher doses of GDMT post-implant, with consequential survival benefit [121–123]. This is particularly relevant to the angiotensin receptor neprilysin inhibitors (ARNIs) such as sacubitril/valsartan (the first drug class to show prognostic benefit in HF for over a decade) [124], as they have a relatively potent effect on blood pressure. Whereas receiving ARNI pre-implant has only shown a limited impact on LVRR in CRT patients [125], observational evidence of CRT non-responders suggests that introducing an ARNI *post*-CRT does impact clinical outcomes [126,127]. Specifically, Sardu et al. compared the clinical outcomes of 418 CRT non-responders, 106 of whom had recently started sacubitril/valsartan versus 312 who had never received an ARNI [127]. At 1-year follow-up, they found 37/106 (34.9%) of ARNI users had become CRT responders compared to 20/312 (6.4%) of non-ARNI users (HR 8.088; 95% CI 4.41–14.84; *p* = 0.000), with the greatest impact seen after 6 months of starting treatment. The same group also found glucagon-like peptide 1 receptor agonist (GLP-1 RA) use in diabetic HF patients was associated with a 3.7-fold increase in CRT response at 1-year follow-up (HR 3.707; 95% CI 1.226–14.570; *p* = 0.026) [128], which is particularly relevant given recent interest in this drug class [129,130], and the growing prevalence of diabetes among HF patients [5]. These results add to a preexisting abundance of data supporting the clinical benefits of GDMT and co-morbidity management in HF, further signaling the importance of optimizing medical therapy after CRT implants.

### 4.4. Optimizing arrhythmia after CRT

Arrhythmia is extremely prevalent among the CRT population. Fortunately, CRT implantation makes stricter arrhythmia management possible, for example, through facilitating AVNA in AF, or via the up-titration of negatively chronotropic anti-arrhythmic medication. Most data on arrhythmia management in CRT have come from sub-analyses of HF trials, with very few studies dedicated to CRT recipients. However, from the latest data, some prominent themes have emerged.

Firstly, it is important in CRT to aim for as high a BiVP percentage as possible to achieve the greatest prognostic benefit [131,132]. Given that arrhythmia is the leading cause for low BiVP [133], this means being relatively proactive with arrhythmia management in CRT compared to the general population (the latter of whom also have growing research to support early arrhythmia control management) [134]. Even in those who have significantly remodelled, removing BiVP can reverse any positive gains made and thus, much like with GDMT, high BiVP percentages should remain uninterrupted long term to maintain their clinical benefit [135,136].

The issue therein lies with which exact management strategy to deploy, as this depends on the type and nature of the arrhythmia. For arrhythmias relatively straightforward to ablate, such as non-AF supraventricular tachycardias, ablation is a favorable, relatively definitive treatment strategy [137]. Even for harder to treat arrhythmias, such as ventricular tachycardia (VT) and premature ventricular contractions (PVCs); however, ablation has still proven beneficial in CRT. Most recently, a retrospective analysis of 64 CRT patients with low BiVP secondary to PVCs or VT found that a higher proportion had improved NYHA class (*p* = 0.003) and reached BiVP ≥98% (*p* < 0.001) after ablation than from medical therapy alone [138]. And, although challenging, even multifocal PVC ablation is becoming increasingly possible with advances in image-guidance and pace-mapping software [139].

The data, however, are more complex to decipher when it comes to AF, the commonest arrhythmic cause of non-response in CRT [4,133]. AF is a dynamic condition, most conventionally commencing as paroxysmal (pAF) before evolving into persistent (persAF) and eventually permanent AF. AF ablation also has relatively low success rates, especially in the HF population. Thus, there is no one-size fits all approach to its management, although the overall goal remains consistent: to minimize its interference with BiVP.

Given its progressive nature and with a higher chance of success from ablation in pAF versus persAF [140], there is increasing support for more proactive rhythm-control management strategies in HF and CRT populations. The recent CASTLE-HTx trial was particularly influential, as this enrolled end-stage HF patients, often deemed too unwell for ablation and usually excluded from clinical trials [141]. Here, 194 patients with LVEF ≤ 35% and symptomatic AF were screened from HTx and LVAD referrals to be eligible for randomization to either catheter ablation and GDMT or medical therapy alone. Of note, 38% of participants had a CRT-D in situ. Ultimately, the trial was stopped early for efficacy, with ablation conferring a significant mortality benefit (HR 0.29; 95% CI 0.12–0.72) and reduction in composite endpoint of all-cause mortality, LVAD implant, or HTx (HR 0.24; 95% CI 0.11–0.52; *p* < 0.001) over 18 months. Ablation recipients also had a larger improvement in LVEF at 6 months (+6.7 ± 6.5% vs +1.2 ± 6.4%; 95% CI 3.5–7.5%) and at 12 months (+7.8 ± 7.6% vs +1.4 ± 7.2%; 95% CI, 4.1–8.7%). The results forced the cardiology community to rethink their approach to end-stage HF patients and caused some to hail AF ablation as the *‘fifth pillar’* [142] of HF treatment alongside GDMT. Although we have no RCTs of AF ablation versus medical therapy in a dedicated CRT population, much like in CASTLE-HTx, similar trials supportive of AF ablation in HF tend to include a significant proportion of participants with CRT, and there is no indication that catheter ablation is inferior to medical management of AF in CRT [143–145]. This is corroborated by a small CRT-specific cohort study of 38 non-responders, in which AF ablation led to significant improvements in BiVP% (*p* < 0.0001), NYHA class (*p* < 0.0001), and LVEF (*p* < 0.0225) from baseline [146].

CRT patients also have the option of AVNA, a procedural form of AF rate-control with equally supportive evidence over medical therapy alone. Most recently, the APAF-CRT mortality trial demonstrated mortality benefit from AVNA when in combination with de-novo CRT in permanent AF patients, compared to medical therapy alone (estimated all-cause mortality at 4 years 14% vs 41%; HR 0.26; *p* = 0.004) [147], but this does not tell us whether AVNA is superior to AF ablation in those who already have a CRT in situ. CRT patients who undergo AVNA cannot receive sLVP or have AVD optimization, both of which we have shown can influence clinical outcomes. Restoring the sinus rhythm also restores the mechanical atrial systole, which can contribute up to 30% of total cardiac output [148], but AF ablation’s relatively low procedural success rates make it uninviting for many patients and clinicians. An RCT is currently underway to directly compare AF ablation versus AVNA, which should address the relative importance of restoring sinus rhythm versus prioritizing BiVP in CRT patients [149].

### 4.5. CRT optimization clinic

The most logical way to holistically manage all aspects of post-CRT care is through a dedicated CRT optimization clinic. There are no RCTs evaluating such clinics, but older observational studies do support multi-disciplinary (MDT) CRT care pathways [150]. In a paper still extensively cited today, Mullens et al. described their insights from having a dedicated optimization clinic for CRT non-responders [8]. Causes of non-response, from most to least common, included suboptimal AV timing, arrhythmia, anemia, suboptimal medical therapy, and baseline narrow QRS. Patients who had subsequent alterations made to their device settings or medical therapy as a result had a lower risk of long-term adverse events than patients who had no changes made (13% vs 50%, *p* = 0.002), favoring a clinic for non-responders. A subsequent study by the group, not limited to non-responders, also observed less adverse events from having a dedicated post-CRT clinic vs standard care (14% vs 53%; *p* < 0.001), as well as a greater improvement in LVEF at follow-up (+11 ± 7% vs +7 ± 9%; *p* = 0.01) [151]. The most common interventions made at the clinic were the up-titration of GDMT (64%) and echocardiography-guided AVD optimization (50%), highlighting the importance of a broad, holistic approach to post-CRT care.

More modern data on the topic has either focused on the combined benefit of pre- and post-CRT implant care pathways [152–154] or observed outcomes in single groups without baseline-matched controls [155,156]. These remain broadly supportive of MDT-guided optimization for *all* CRT recipients, with multiple interventions made as a result [156], but cast some doubt over the routine need for echocardiography-guided optimization [155]. In addition, all studies focus on isolated follow-up visits rather than the impact of continuous monitoring, failing to account for the dynamic nature of CRT response in the way that inbuilt device algorithms can. Given the resource-heavy nature of CRT optimization clinics, more robust data is required to advocate for a more frequent clinic-based model for post-CRT care going forward.

## 5. Conclusion

CRT is a highly beneficial treatment for a large proportion of dyssynchronous HF patients but yields disappointing results for a significant minority. Optimizing its potential requires a multifaceted approach at all stages of the patient journey. Key aspects of patient selection include a deeper understanding of QRSd and morphology, and the use of multimodal imaging. For implantation, an expanding number of options are now available, allowing for patient-specific approaches to treatment. After implant, care must remain continuous to reflect the dynamic nature of CRT response, including the use of device-based algorithms alongside MDT-based arrhythmia and medication optimization. Most importantly though, a clearer understanding of ‘CRT response’ is required, to ensure we are not devaluing the true benefits of CRT in its current form.

## 6. Expert opinion

We have explored the latest research into optimizing CRT response, but the question remains – what should the aims of resynchronization therapy be? Traditional definitions of CRT response have potentially hampered our understanding of what can be objectively achieved from CRT, with a focus on echocardiographic rather than QOL-based outcomes. This has led to misconceptions about the efficacy of CRT, leading to its underutilization in suitable patients.

In addition, research into patient selection has exposed the vulnerability of current guidelines, which oversimply the nature of dyssynchronous HF, leading to CRT implantation in unsuitable candidates. Future guidelines will need to be adapted to match the latest plethora of data on this topic and move toward a precision medicine approach. In particular, future guidelines may wish to adopt modified QRSd (QRSd/LVEDV) as a CRT indicator for all patients, or reduce the absolute QRSd cutoff for women only (as per several recent CRT trials) [79,111]. More data, in this previously under-researched field, are needed to understand sex-specific characteristics in cardiology, as we are likely underutilizing CRT in women – a common theme in all aspects of female cardiology treatment worldwide [157].

Conjuring great excitement in the pacing community is CSP or optimized-CRT as alternatives to cCRT. However, the relative novelty of these techniques has meant the data is currently limited by several factors. Firstly, the steep learning curve has meant relatively high implant failure rates. For example, in the LEVEL-AT trial, LBBP rendered an 82% implant success rate and HBP had just 57% implant success [158]. This has likely caused biased against CSP in the earliest trials, and it will be interesting to see what happens as experience with CSP widens. Secondly, many CSP trials have used either HBP or LBBP rather than a singular modality. This, in conjunction with high crossover rates, makes the data difficult to interpret, especially in small cohorts. Lastly, as with any novel treatment, we are yet to know the full long-term impact of CSP and optimized-CRT; high pacing thresholds from HBP are a particular worry, and the current evidence-base for CSP lead extraction is limited. Nonetheless, with more RCTs in the pipeline, we see physiological pacing playing an increasingly greater role in dyssynchronous HF management going forward.

The rise of AI has been another exciting addition to our understanding of CRT response. In particular, AI-led ECG interpretation offers the advantage of being reproducible and easy to automate, addressing the subjectiveness and intervariability of clinician-led ECG interpretation [159]. In future practice, we envisage AI tools being used at PAC, taking multiple parameters into account including ECG data, imaging, and biomarkers to predict individual patients’ responses to CRT. However, the main limitation of current AI-ECG trials is the use of data which predates the introduction of novel HF medications such as ARNIs and SGLT2 inhibitors, both of which have independently improved long-term outcomes for modern-day HF patients [124,160,161]. Newer, prospective trials are sorely needed to test these prediction methods in contemporary HF populations. In addition, individual prediction methods need to be tested against one another, as there are currently risks there being too many ML-based CRT prediction scores for clinicians to choose from [24,25,162,163], ultimately defeating the point of having an objective scoring method.

Looking further into the future, we envisage AI tools being used alongside knowledge gained from in silico research to allow for truly personalized medicine. This would be achieved via ‘virtual pacing,’ whereby a digital version of a patient’s heart is paced prior to implantation, to test the acute impact of different pacing modalities and in turn predict any long-term benefit from CRT in that individual. A recent study demonstrated that acute reductions in septal-to-lateral myocardial work difference could be assessed in response to simulating BiVP in ‘digital twin’ heart models, created from the baseline echocardiographic data of 45 CRT recipients [164]. In turn, both baseline septal-to-lateral myocardial work and change in work upon virtual pacing were significantly associated with the degree of LVRR in the real-life patients at 6 months (*r* = −0.60; *p* < 0.001 and *r* = 0.62; *p* < 0.001, respectively), a finding which has been shown in other real-world studies [38]. Virtual pacing in this manner has advantages over temporary ‘in vivo’ pacing intra-procedure of being less time-consuming, cheaper, and easier to deploy on a large scale clinically. In practice, clinicians could review what has worked for their patient ‘digitally’ in CRT PAC, before deciding on a final treatment plan. By using such a personalized approach, one would expect a consequential improvement in long-term outcomes from CRT.

Not to be forgotten is post-implant care, where one of the biggest areas for growth has been device-based optimization algorithms. Despite so far only showing modest impacts on clinical outcomes, algorithmic CRT optimization is certainly here to stay. It has the major advantages of being dynamic and less time-consuming than traditional face-to-face echocardiography-based CRT optimization, which as a result of being so labor-intensive has had relatively poor uptake globally [165]. In addition, although at a higher upfront cost, devices with advanced algorithms appear to be cost-effective long term via slowing battery depletion [111], reducing hospitalizations [166,167] and improving survival [168]. Looking forward, we await the results of two upcoming trials from device companies to see if their CRT algorithms have any significant impact on long-term clinical outcomes [105,106].

Overall, it is impossible to predict which factors will come to have the most influence on CRT optimization long term, and the concomitant nature of pre-, peri- and post-implant research hinders a clear delineation of relative impact. With the data currently available, we advise clinicians to approach CRT care with a greater understanding of its complexity than traditionally expected; in Table 1 we have outlined the different aspects to consider at each stage of the process, as guided by the research we have presented. Looking forward, once patient selection and pacing techniques are finely tuned, the ultimate goal will be to provide truly bespoke CRT, maximizing each individual’s potential from resynchronization therapy. Whether this will truly happen, or be an idea better in theory than in practice, remains to be seen.

**Table 1.** Interventions to consider at each stage of the CRT pathway.

When	Intervention	Considerations	Latest insights
Pre-implant	CRT PAC	MDT pre-assessment	Cost- and clinically effective way of ensuring adherence to current CRT guidelines and an opportunity to optimize other aspects of HF care, including arrhythmia, anemia and GDMT
	12-lead ECG	QRSd	QRSd/LVEDV is a more accurate metric than raw QRSd; consider a lower QRSd cutoff (≥120-130 ms) for women
		QRS morphology	Patients with LBBB and IVCD are more likely to respond to cCRT; IVCD is less likely to respond to CSP; typical RBBB with QRSd <150 ms is unlikely to respond to any current form of CRT
		PR interval	Non-LBBB patients with a normal PRi are less likely to respond to cCRT; prolonged PRi is a strong predictor of CRT respond, including to CSP
	AI-ECG	QRSarea	Low QRSarea (calculated by VCG) is less likely to respond than high QRSarea
		Machine learning	Can use a ML algorithm to predict chance of CRT response from baseline ECG
	Blood test	Novel biomarkers	Blood tests for RyR1 glycation and micro-RNAs may be considered to predict CRT response, but are not widely available
	Echocardiography	LVEF	No new research to dispute the current cutoff of LVEF ≤ 35% for CRT (unless for a non-HF indication such as likely high burden RV pacing)
		Mechanical dyssynchrony	Variable data to support its routine use in vetting CRT referrals, and certainly shouldn’t be used in place of ECG measures of dyssynchrony
	CMR	Scar location	Patients with septal scar are less likely to respond to BiVP or CSP; may require CSP-optimized CRT instead
		HF etiology	CMR can help diagnose underlying HF etiology. Nonischemic cardiomyopathy is more likely to respond to CRT than ischemic cardiomyopathy
		Machine learning	Can use a ML algorithm (± echocardiography data) to predict chance of CRT response from baseline imaging
	Virtual pacing	In silico testing	Can predict the likely degree of LVRR from acute responses to virtual CRT computational models
Peri-implant	cCRT	Image-guided epicardial pacing	Favorable to avoid scar, but in practice too challenging to employ routinely
		Multisite pacing	Lacks convincing evidence to support its routine use
	Endocardial LV pacing	Lead-based or leadless	Lead-based endocardial LVP is not recommended; leadless LVP is a feasible and safe option where endovascular CRT is not possible or there is cCRT non-response.
	CSP-CRT	HBP	HBP is at least non-inferior to BiVP but limited by high lead thresholds and implant success rates. Longer term data is awaited
		LBBP	LBBP is comparable to BiVP in small trials and has greater implant success rates than HBP. Again, longer term data is awaited (especially regarding the ease of lead extraction) and more data is required for it to overtake BiVP as a first-line CRT method
		CSP-optimized CRT	Impressive results from small observational trials so far; worth considering in cCRT or CSP non-responders. Needs much more data to become a first-line therapy
Post-implant	Device settings	Echocardiography-guided optimization	Achieves acute hemodynamic benefit, but consistently no evidence of any long-term impact
		Device-based algorithms	Weak impact on long-term outcomes so far, but positive impacts on echocardiographic outcomes, especially from high sLVP% when compared to BiVP. Currently cost-effective and addresses the dynamic nature of LV remodeling. Several relevant trials to be published soon
	GDMT	HF medication optimization	Up-titrating HF medication is more possible after CRT implantation and impacts long-term clinical outcomes. Important to add an ARNI and SGLT2i where possible
	Arrhythmia	Ablation or medical therapy	Proactive, early ablation-based treatment where possible. Aim for BiVP as close to 100% as possible; low BiVP, even if the LV has reverse remodeled, is detrimental to long-term clinical outcomes
	CRT optimization clinic	MDT follow-up	Likely useful for all CRT recipients to attend a face-to-face MDT follow-up once, if only to up-titrate HF medication and address arrhythmia; evidence is less supportive of the need for regular repeat echocardiography-based optimization, especially with the increasing availability of real-time device-based algorithms

AI, artificial intelligence; ARNI, angiotensin receptor/neprilysin inhibitor; BiVP, biventricular pacing; CMR, cardiac magnetic resonance; CRT, cardiac resynchronization therapy; CSP, conduction system pacing; cCRT, conventional CRT; ECG, electrocardiography; HF, heart failure; GDMT, guideline-directed medical therapy; HBP, His-bundle pacing; IVCD, intraventricular conduction delay; LBBB, left bundle branch block; LBBP, left bundle branch pacing; LV, left ventricle/ventricular; LVEDV, LV end-diastolic volume; LVEF, LV ejection fraction; LVP, LV pacing; LVRR, left ventricular reverse remodeling; MDT, multidisciplinary team; ML, machine learning; PAC, pre-assessment clinic; PRi, PR interval; QRSd, QRS duration; RBBB, right bundle branch block; RV, right ventricle/ventricular; RyR1, Ryanodine Receptor 1; SGLT2i, sodium-glucose co-transporter-2 inhibitor; sLVP, synchronized LVP.

## Article highlights

- We are likely underselling the efficacy of conventional cardiac resynchronization therapy (cCRT) with an outdated understanding of ‘CRT response.’
- Patient selection remains crucial to optimizing CRT outcomes. Current guidelines are too broad, lacking the sophistication to match the true variability of heart failure (HF) patients. In particular, not all QRS durations ≥130 ms are equal, especially in women or those of small body habitus.
- Artificial intelligence (AI) is increasingly helpful in understanding which baseline characteristics best predict outcomes from CRT.
- Newer, non-epicardial forms of CRT are gaining traction at a fast pace, in particular conduction system pacing (CSP). There is not yet enough research to know if this will overtake cCRT as a first-line treatment for dyssynchronous HF. We also see CSP being used in conjunction with cCRT in the form of ‘CSP-optimized CRT’
- Traditional echocardiography-based optimization strategies post-implant lack compelling evidence and are time-consuming. Instead, device-based algorithms allow for real-time alterations to device settings to match the dynamic nature of CRT response, but so far lack convincing outcome data to prove they make any significant long-term impact.
- Optimizing non-device care holistically, including arrhythmia and medication management, remains vital to maximizing outcomes from CRT.
- The ultimate future goal is for personalized care, using in silico testing and artificial intelligence to guide individualized CRT pathways.
